# Promotion of In Vitro Osteogenic Activity by Melt Extrusion-Based PLLA/PCL/PHBV Scaffolds Enriched with Nano-Hydroxyapatite and Strontium Substituted Nano-Hydroxyapatite

**DOI:** 10.3390/polym15041052

**Published:** 2023-02-20

**Authors:** Georgia-Ioanna Kontogianni, Amedeo Franco Bonatti, Carmelo De Maria, Raasti Naseem, Priscila Melo, Catarina Coelho, Giovanni Vozzi, Kenneth Dalgarno, Paulo Quadros, Chiara Vitale-Brovarone, Maria Chatzinikolaidou

**Affiliations:** 1 Department of Materials Science and Technology, University of Crete, 70013 Heraklion, Greece; 2 Research Center E. Piaggio and Department of Information Engineering, University of Pisa, 56126 Pisa, Italy; 3 School of Engineering, Newcastle University, Newcastle upon Tyne NE1 7RU, UK; 4 FLUIDINOVA S.A., 4475-188 Maia, Portugal; 5 Department of Applied Science and Technology, Politecnico di Torino, 10129 Turin, Italy; 6 Foundation for Research and Technology Hellas (FORTH)-IESL, 70013 Heraklion, Greece

**Keywords:** composite scaffolds, bone tissue engineering, extrusion, fused deposition modeling (FDM), 3D printing, PLLA, PCL, PHBV

## Abstract

Bone tissue engineering has emerged as a promising strategy to overcome the limitations of current treatments for bone-related disorders, but the trade-off between mechanical properties and bioactivity remains a concern for many polymeric materials. To address this need, novel polymeric blends of poly-L-lactic acid (PLLA), polycaprolactone (PCL) and poly(3-hydroxybutyrate-co-3-hydroxyvalerate) (PHBV) have been explored. Blend filaments comprising PLLA/PCL/PHBV at a ratio of 90/5/5 wt% have been prepared using twin-screw extrusion. The PLLA/PCL/PHBV blends were enriched with nano-hydroxyapatite (nano-HA) and strontium-substituted nano-HA (Sr-nano-HA) to produce composite filaments. Three-dimensional scaffolds were printed by fused deposition modelling from PLLA/PCL/PHBV blend and composite filaments and evaluated mechanically and biologically for their capacity to support bone formation in vitro. The composite scaffolds had a mean porosity of 40%, mean pores of 800 µm, and an average compressive modulus of 32 MPa. Polymer blend and enriched scaffolds supported cell attachment and proliferation. The alkaline phosphatase activity and calcium production were significantly higher in composite scaffolds compared to the blends. These findings demonstrate that thermoplastic polyesters (PLLA and PCL) can be combined with polymers produced via a bacterial route (PHBV) to produce polymer blends with excellent biocompatibility, providing additional options for polymer blend optimization. The enrichment of the blend with nano-HA and Sr-nano-HA powders enhanced the osteogenic potential in vitro.

## 1. Introduction

Bone defects result from trauma, bone diseases, and cancer, and their functional restoration is a clinical challenge [1]. The gold standard methods to treat bone defects include the implantation of autografts, allografts, or xenografts. However, these therapeutic approaches have many disadvantages, including risk of infection, limited availability, and adverse immune responses. To overcome these limitations, bone tissue engineering (BTE) presents an attractive strategy to treat bone defects using natural or synthetic materials to create the appropriate environment for the enhancement of bone tissue regeneration [2].

In the last several decades, 3D printing techniques (i.e., the application of Additive Manufacturing technologies to the fabrication of scaffolds by the deposition and assembling of living and/or not living biomaterials with an established organization [3]) have replaced conventional technologies in the fabrication of BTE scaffolds thanks to their precise and repeatable control over the scaffold macro- (overall shape and size) and micro- (pore size) architecture [4,5]. Among the various techniques available [6,7], fused deposition modeling (FDM) is one of the most commonly used fabrication methods in the BTE field since it can process materials with mechanical properties close to those of the natural tissue [8,9]. FDM uses a thermoplastic filament heated around its softening point and extruded from a nozzle onto a printing bed to produce a 3D object layer-by-layer [10]. The filament is typically produced through Hot Melt Extrusion (HME), an industrial process in which a softened material is forced to pass through a die thanks to an upstream pressure. The HME machine exploits the rotation of one (single screw extruder) or two (twin screw extruder) parallel screws placed inside a heated barrel to convey the solid feedstock (usually in pellets or in powder form) from a hopper upstream, where it is melted and extruded through the die [11]. Through HME it is possible to produce filaments starting from single polymers, like poly(lactic acid) (PLA) [12], poly(ε-caprolactone) (PCL) [13], poly(methyl methacrylate) (PMMA) [14], and polycarbonate (PC) [15].

One of the most popular biomaterials used in medical devices is PLA, a biocompatible thermoplastic polyester with low toxicity [16] and compressive strength (2–39 MPa) similar to the trabecular bone tissue (2–12 MPa) [17]. Although PLA has good mechanical properties for BTE applications, it is still lacking ductility and bio-functionality showing low biological activity [18], which limits its applications. PCL is another FDA approved and biodegradable polyester with a low melting point, widely used in BTE due to its biocompatibility, controllable degradation rate, and excellent mechanical properties [19]. However, PCL constructs lack attachment signals leading to decreased cell adhesion and thus bioactivity [19]. Lastly, poly(3-hydroxybutyrate-co-3-hydroxyvalerate) (PHBV) is a poly(hydroxyalkanoate)-type of polymer produced by bacteria and has been shown to promote favorable bone tissue responses when implanted in vivo [20]. This polymer is also biodegradable, non-toxic and biocompatible but lacks mechanical strength, in contrast to the other two polymers, limiting its application in BTE [21]. In order to overcome the limitations of each material when used separately, HME can be used to blend these different polymers and create a composite filament with tailored physical properties such as degradation rate, and mechanical characteristics (e.g., Young modulus) [22,23,24]. Biodegradation is another crucial parameter for BTE applications, as degradable implants eliminate the need for a second surgical event for their removal. Degradable devices can also carry drugs and growth factors that can be gradually released during biodegradation to accelerate bone healing [25]. As degradation evolves, the applied loading can be slowly transferred to the healing bone at a rate that is controlled during engineering of the polymeric material.

Furthermore, the inclusion of different inorganic phases, including nano-hydroxyapatite (nano-HA) or tricalcium phosphate, can impart the printed scaffold with enhanced biological properties [24,26,27]. Nano-HA is the main inorganic component of bone and is widely used in BTE integrated into 3D matrices due to its biocompatibility and osteoconductive properties [28]. Similarly to the commercially available nano-HA, the apatite of the human body increases the surface activity of the materials that can be incorporated [29]. An additional advantage of nano-HA is its neutralizing properties, as it can react with the polymeric acidic by-products, thus increasing the biological activity of the composites [30].

These are all key factors to consider nano-HA for applications in BTE [31,32,33]. Common combinations of materials include poly(vinyl alcohol) (PVA)/beta-tricalcium phosphate [34], PCL/HA [35] and PLA/HA [36]. In a recent study, Zavrel et al. explored the use of HME to obtain a composite filament from PCL and HA [37]. The authors investigated the effect of both PCL particle size and HA content (up to 40 wt%) on extrudability, filament homogeneity and FDM printability, as well as the cytocompatibility of the printed scaffolds in terms of cell viability, adhesion, and proliferation. Due to the difference in particle size, PCL pellets and HA powder were added sequentially inside the feeding channel of the extruder. As an alternative to this solution, Kim et al. created a pre-mixed pellet feed by first dissolving PCL and HA in N,N-dimethylformamide (DMF). The two solutions were then mixed by stirring until the DMF was completely evaporated. After solidification, the PCL-HA composites were cut into small pieces to obtain pellets for the HME process [38]. On a similar note, Corcione et al. [6] used a twin-screw extruder to blend PLA with nano-HA. To ensure proper mixing of the two materials, PLA pellets were coated with the nano-HA powder using a rotomolding machine. Thermogravimetric analysis (TGA) showed the same amounts of nano-HA used for the production of the composites, while rheological analysis demonstrated that the printing temperature of PLA is not influenced by the presence of nano-HA [39].

The incorporation of physiologically-occurring-in-the-human-body trace elements into materials aiming to promote their biological activity is an area of growing interest [40]. The addition of elements such as strontium (Sr), magnesium, or selenium into 3D matrices enhances their osteogenic properties [26]. Particularly, Sr was reported more than half a century ago to favor the bone metabolism by increasing new bone formation and reducing bone resorption. Strontium renelate is used as a commercial product for the treatment of postmenopausal osteoporosis due to its potential to stimulate the osteoblasts’ viability and differentiation and to hinder the osteoclasts proliferation and activity in a dose-dependent manner [41]. The substitution of calcium by strontium in the HA crystal structure has been widely reported due to their chemical analogy since both are divalent cations with similar ionic radii. A substitution degree at a concentration less than 10% has been described to enhance the dissolution kinetics of HA and therefore its degradability [42]. An enhanced dissolution of the inorganic phase leads to a higher concentration of ions exerting a biological effect, expecting an amplification of the osteogenic potential [36]. The substitution of calcium ions by strontium ions in HA has been reported in the context of calcium phosphate ceramics, coatings, and bioactive bone cements, showing an increase of their osteogenic potential in vitro and in vivo [43]. The composition and morphology of the strontium-substituted nano-HA is expected to affect the ion release rate, which in turn influences the cellular responses [44]. In addition, the particle size and the surface properties of the inorganic phase influence their incorporation within polymer matrices, thus, changing the degradation and mechanical properties of the composites [36]. Strontium-substituted calcium nano-HA has been recently reported as a component of composite scaffolds based on gelatin [45], poly(hydroxyalkanoates) [46], poly(lactide-co-glycolide) [47], or PCL-poly(ethylene glycol) [48]. These composite scaffolds have been used in BTE, indicating an enhancement of osteogenic cell differentiation and reduction of the osteoclastic activity. Only a few reports propose strontium-substituted HA in pure PLA-based composites for potential BTE applications [49,50,51].

Here we propose, prepare, and evaluate a novel formulation that builds on and takes a step forward from the current literature by combining three different polymeric components, namely: (i) PLLA, to impart mechanical strength and improve extrudability; (ii) PCL, to enhance the filament elasticity; and (iii) poly(3-hydroxybutyrate-co-3-hydroxyvalerate) (PHBV), to increase the material biological affinity. Furthermore, the basic PLLA/PCL/PHBV blend was enriched with two inorganic phases, nano-HA and strontium-substituted nano-HA (Sr-nano-HA), to increase the osteogenic potential of the printed BTE scaffolds. The optimal printing parameters for the FDM technology such as printing speed, nozzle and bed temperature were optimized for each composition and used to fabricate cylindrical scaffolds for in vitro biological evaluation. The filaments and the scaffolds were assessed for cytotoxicity using MC3T3-E1 pre-osteoblastic cells. The cell morphology was investigated by means of scanning electron microscopy. Moreover, the osteogenic differentiation potential of pre-osteoblastic cells cultured within 3D printed polymer blend and composite scaffolds was evaluated to assess their suitability for bone tissue regeneration.

## 2. Materials and Methods

### 2.1. Polymer Raw Materials

PLLA (PURASORB PL38) was obtained from Corbion (Amsterdam, Netherlands). PCL (average M_w_ 80,000) and PHBV (PHV content 8 mol%) were obtained from Merck (Darmstadt, Germany).

### 2.2. Preparation of Nano-HA and Sr-nano-HA

The nano-HA materials used in this study were prepared using an existing protocol [52]. Briefly, aqueous solutions of Ca^2+^ and PO_4_^3−^ ions were fed into the NETmix^®^ reactor [53] under controlled pH conditions. To obtain the Sr-nano-HA material, an additional source of Sr^2+^ was also added. The samples were obtained by wet chemical precipitation and the resulting slurries were continuously washed to remove residual ions and further concentrated to obtain suspensions with a final concentration of 15.5 ± 1.0 wt%, with 100% of Ca^2+^ in nano-HA and 50% of Sr^2+^ substitution in case of the Sr-nano-HA. The chemical characterization of the Sr-nano-HA materials has been previously reported [36] confirming the effective incorporation of Sr^+2^ in nano-HA lattice without decomposition of the nano-HA phase.

### 2.3. Preparation of Polymer Blend and Composite Filaments

Filaments of a three-component PLLA/PCL/PHBV (90/5/5 wt%) polymer blend (designated as blend), were prepared using extrusion (Rondol Microlab 10 mm twin screw extruder), as described in a previous study [54], which explored the use of PCL and PHBV in combination with PLLA towards the evaluation of the thermal, mechanical and processing properties of the blends. Briefly, the process involved mixing of PLLA, PCL and PHBV pellets, and the mixture was used as feedstock for the extruder to produce the blend. The specific ratio of 90/5/5 wt% was selected based on the mechanical robustness of the blend composition and its extrudability without thermal or mechanical degradation of its components [54]. After the filaments had been extruded, they were cut to lengths of approximately 0.5 mm using a pelletizer (Rondol). In addition, two enriched blends were produced incorporating nano-HA and Sr-nano-HA powders. The powders were incorporated by pre-mixing the PLLA pellets with the 2.5% nano-HA or Sr-nano-HA paste. The concentration of nanoparticles was selected such that the mechanical properties of the composite materials would not be significantly reduced through the inclusion of the inorganic phases. This mixture was left at room temperature for 12 h prior to incubation at 50 °C overnight, drying the paste onto the outer surfaces of the PLLA pellets. The nano-HA or Sr-nano-HA coated PLLA pellets were then combined with the other constituents of the blend (PCL and PHBV) and thoroughly mixed manually. The mixture of pellets was then extruded using the same conditions as for the non-enriched blend. Table 1 reports the diameter of the produced filaments, based on a series of measurements along the length of each filament.

Pellets of the produced filaments were assessed for degradation by incubating in phosphate buffer saline (PBS) at 50 °C for eight weeks, with mass loss recorded weekly, a weekly change in PBS and pH measurement to determine the presence of any acidic degradation by-products.

### 2.4. Preparation of Scaffolds

#### 2.4.1. Selection of the Optimal Printing Parameters

A Prusa i3 Rework FDM printer was used for all printing experiments. The printer was equipped with: (i) a 0.4 mm brass nozzle; (ii) a textured printing plate; and (iii) an enclosure to keep the temperatures more uniform during printing. For each filament composition, a different heating module (i.e., nozzle, heat break, internal Teflon tube) was used to avoid any cross-contamination between the materials.

Preliminary extrusion experiments were used to evaluate the printing temperatures for each filament composition. The extrusion temperature was calibrated by extruding a fixed amount of material at temperatures in the range of 190–220 °C in 10 °C steps whilst controlling the quality of the extruded strand. Temperatures identified as being too low consisted in those for which it was difficult to extrude the material through the nozzle (i.e., clinking sounds could be heard during extrusion); on the other hand, if fumes could be seen during extrusion, then the temperature was deemed too high. For the intermediate temperatures, the quality of the extruded strand was evaluated using an optical microscope (Leica DM6 M).

The mean diameter values were specified in the slicing software by setting the filament diameter parameter for all printing tests (Table 1). Other relevant parameters (i.e., bed temperature, printing speed) were chosen directly by printing simple shapes (i.e., cylinders of 5 mm in diameter, 1 mm in height and no pores) and tuning them based on the adhesion to the printing plate. In general, a combination of lower speeds and higher bed temperatures was preferred to increase the material adhesion.

The set of parameters used for scaffold printing is summarized in Table 2.

#### 2.4.2. Scaffold Design, Printing and Characterization

**Figure 1:**
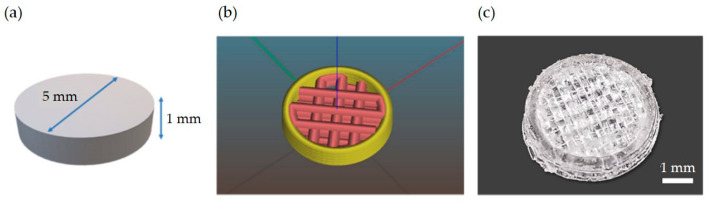
Cylindrical scaffold overall dimensions (**a**); the internal topology selected for the biological evaluation (**b**); an example of the final printed scaffold produced from the blend filament (**c**).

A cylindrical scaffold shape of 5 mm diameter and 1 mm height was produced for all biological experiments (Figure 1). This specific geometry was chosen so that the scaffold could fit inside a standard well of a 96 multi-well plate. Regarding the internal topology, a line infill with a 50% porosity was selected for all layers except the first one, which was set to full (i.e., without any macroscopic pores), so that cells could be seeded from the top of the scaffold without leaving it and adhering to the tissue culture plastic. As a result, the overall porosity of the whole scaffold resulted as 40%.

All scaffolds were printed using the optimized parameters from the preliminary experiments of Section 2.4.1. After printing, the geometry and print fidelity were characterized by manually measuring the diameter and height with a digital caliper. Moreover, the scaffold porosity was evaluated by weighing ten scaffolds printed from each material. The porosity *P* (expressed as a percentage) was then computed by considering the following relationship:

$$P=\frac{V_{tot}-V_{pol}}{V_{tot}}$$

where *V_tot_* is the total external volume of the scaffold and *V_pol_* the volume of the polymeric part in the porous scaffold (both measured in [cm^3^]). By considering that V = m/ρ (ρ the polymer density in [g/cm^3^], m the mass in [g]), we can write:

$$P=1-\frac{m_{pol}}{m_{tot}}$$

Here, *m_pol_* is the measured mass of the scaffold and *m_tot_* is the overall mass of a scaffold with the same external dimensions but no porosity. The latter value was estimated knowing the scaffold dimensions and using an estimated density of 1.2 g/cm^3^ for all materials. This value was approximated by considering the mean density of the single polymeric components PLLA, PCL, and PHBV weighted for their mass content in the final filament.

The scaffold effective compressive modulus was measured in an unconstrained compression setup using a universal mechanical testing machine (Zwick Roell Z-005). The scaffolds (6 per filament group, n = 18) were tested using a parallel plate geometry by applying a constant deformation at 1% strain per second until a deformation of 10% strain was attained. The stress was estimated by dividing the measured force by the apparent area, which was calculated using the Feret diameter of the scaffolds. Data were then analyzed using GraphPad Prism software to find the effective compressive modulus by evaluating the slope of the initial part of the stress-strain curve using linear regression.

The scaffold morphology was assessed with scanning electron microscopy (SEM) using the TM3030 desktop SEM (Hitachi, Ibaraki Prefecture, Japan), using an accelerating voltage of 15 kV. The same equipment was used to detect the presence and distribution of the nanoparticles in the printed 3D scaffolds (n = 3). Using the equipment’s Energy Dispersive X-Ray Spectroscopy (EDS) module (Bruker, Coventry, UK), it was possible to identify the presence of Sr^2+^ within the inorganic phase and also the base composition of the scaffold. The quantification of elements and their mapping was performed with the Quantax software provided by Hitachi. Values for the concentration of each element (atomic weight percentage—wt.%) were collected and presented as mean ± SD.

### 2.5. In Vitro Biological Evaluation of Pre-Osteoblasts in 3D Composite Scaffolds

Extruded filament pieces of 0.5 mm in length and 3D printed scaffolds produced from the PLLA/PCL/PHBV polymer blends and composites (with nano-HA and Sr-nano-HA) have been evaluated for their cell adhesion and morphology (Section 2.5.3), cell viability and proliferation (Section 2.5.4), and osteogenic differentiation capacity in terms of alkaline phosphatase (ALP) (Section 2.5.5), calcium (Section 2.5.6), and collagen production (Section 2.5.7).

#### 2.5.1. Cell Culture of Pre-Osteoblasts

MC3T3-E1 pre-osteoblastic cells (DSMZ, Braunschweig, Germany, ACC-210) isolated from newborn mouse calvaria have the capacity to differentiate into osteoblasts in vitro [56], and have been used for the investigation of cell adhesion, viability, proliferation, and differentiation of biomaterials. The pre-osteoblastic cells were cultured in complete medium comprising alpha-MEM cell culture medium (PAN-Biotech, Aidenbach, Germany) supplemented with 10% (*v/v*) fetal bovine serum (FBS) (PAN-Biotech, Aidenbach, Germany), 100 μg/mL penicillin and streptomycin (PAN-Biotech, Aidenbach, Germany), 2 mM L-glutamine (PAN-Biotech, Aidenbach, Germany), and 2.5 μg/mL amphotericin (Gibco, Thermo Fisher Scientific, Waltham, MA, USA) in a humidified incubator at 37 °C, 5% CO_2_ (Heal Force, Shanghai, China). The culture medium was replaced twice weekly. The cells were detached using trypsin-0.25% ethylenediaminetetraacetic acid (EDTA) (Gibco, Thermo Fisher Scientific, Waltham, MA, USA). Passages 12–17 of pre-osteoblastic cells were used for the viability and differentiation experiments.

#### 2.5.2. Cell Seeding on Extruded Filament Pieces and Scaffolds

Prior to cell seeding, the polymeric and composite materials in both forms, extruded filament pieces and scaffolds, were sterilized using a two-step approach: (1) immersion in 70% ethanol for 3 min; and (2) 30 min of UV irradiation.

For the cell viability quantification by means of the PrestoBlue^TM^ assay, extruded filament pieces were soaked for 30 min in complete medium in 24-well plates, and, after the removal of the medium, 3 × 10^4^ cells/well were seeded. For the live-dead assay, 5 × 10^3^ cells/well were seeded on the filament pieces. Tissue culture polystyrene (TCPS) was used as the control surface.

For the viability and differentiation experiments on scaffolds, the scaffolds were soaked for 30 min in complete medium in 96-well plates before seeding. After medium removal, 7 × 10^4^ cells/scaffold were pipetted on top of the structures. Cells were diluted in osteogenic medium which contains the complete alpha-MEM medium supplemented with 10 nM dexamethasone (Sigma-Aldrich, Burlington, MA, USA), 10 mM β-glycerophosphate (Sigma-Aldrich, Burlington, MA, USA), and 50 μg/mL L-ascorbic acid 2-phosphate (Sigma-Aldrich, Burlington, MA, USA). All samples were analyzed in quadruplicates of three independent experiments (n = 12).

#### 2.5.3. Adhesion and Morphology of Pre-Osteoblasts within Scaffolds

The morphology of the pre-osteoblastic cells on the scaffolds was monitored by SEM (JEOL JSM-6390 LV, Tokyo, Japan) after 7, 14 and 21 days of incubation. At each time point, cells were rinsed twice with PBS, then fixed with 4% *v*/*v* para-formaldehyde for 20 min at room temperature and dehydrated in increasing concentrations (30% *v*/*v*–100% *v*/*v*) of ethanol. Cell-loaded scaffolds were then dried in a critical point drier (Baltec CPD 030, Baltec, Los Angeles, CA, USA), sputter-coated with a 20 nm thick layer of gold (Baltec SCD 050, Baltec, Los Angeles, CA, USA), and observed under a microscope at an accelerating voltage of 20 kV.

#### 2.5.4. Cell Viability Assessment within Scaffolds

The viability and proliferation of pre-osteoblastic cells was evaluated using the PrestoBlue^TM^ cell viability assay (Invitrogen Life Technologies, Waltham, MA, USA) [57]. The nontoxic metabolic indicator resazurin is modified by the reducing environment of living cells, leading to a red and highly fluorescent product that can be detected photometrically. Cell viability was measured after 3, 7 and 14 days on extruded filament pieces and after 7, 14, and 21 days on scaffolds. Absorbance measurements related to cell viability were performed at 570 and 600 nm using a spectrophotometer (Synergy HTX Multi-Mode Microplate Reader, BioTek, Winooski, VT, USA). The absorbance values were translated to cell number by means of a calibration curve. Quadruplicates of three independent experiments were analyzed (n = 12).

In addition, cell viability within scaffolds was assessed using a live/dead assay (Biotium, Fremont, CA, USA) following the manufacturer instructions. Briefly, the pre-osteoblastic cells were seeded in direct contact with the materials, and after 3, 7 and 14 days of culture they were washed with PBS and then incubated in a working solution containing 2 mM calcein-AM (494 nm excitation/517 nm emission) and 4 mM ethidium homodimer III (EthDIII) (532 nm excitation/625 nm emission) at room temperature for 45 min. The stained cells were observed under an inverted fluorescence microscope. Visualization of the fluorescently labelled cells was performed using the ImageJ software (ImageJ software, sLOCI, University of Wisconsin, Madison, WI, USA).

#### 2.5.5. Alkaline Phosphatase (ALP) Activity Measurement

An enzymatic activity assay was used to measure the levels of ALP activity, an early osteogenic marker produced from the pre-osteoblastic cells cultured on different materials. The ALP activity method is based on the ability of the enzyme to use para-nitrophenyl phosphate (pNPP) as a substrate and hydrolyze it into 4-nitrophenol (pNP) and a phosphate group [58]. Cells were cultured for 7, 14 and 21 days in complete medium, lysed in 200 μL lysis buffer (0.1% Triton X-100 in 50 mM Tris-HCl pH 10.5) after rinsing with PBS, and subjected to two freeze-thaw cycles. Then, 200 μL of a solution containing 2 mg/mL p-nitrophenyl phosphate (pNPP) (Sigma-Aldrich, Burlington, MA, USA) in 50 mM Tris-HCl and 2 mM MgCl2 at pH 10.5 were added to each sample and left at room temperature for 60 min. The reaction was stopped with the addition of 50 μL of 1 N NaOH. The absorbance of the reaction product p-nitrophenol (pNP) was measured at 405 nm in a spectrophotometer (Synergy HTX Multi-Mode Microplate Reader, BioTek, Winooski, VT, USA). The absorbance values were translated to pNP concentrations by means of a calibration curve, and these were normalized to cellular protein determined using the Bradford assay (Applichem, Darmstadt, Germany) in the cell lysates. Quadruplicates of three independent experiments were analyzed (n = 12).

#### 2.5.6. Calcium Production

Calcium production is a late marker of osteogenesis and a key regulator in the formation of the extracellular matrix (ECM). The concentration of the produced calcium was determined by means of staining the supernatants of the cell culture with O-cresol phthalein complexone (CPC) (Biolabo, Les Hautes Rives Maizy, French) [59]. Briefly, 10 μL of the supernatants were mixed with 100 μL of calcium buffer containing 1.7 mol/L amino-2-methyl-2-propanol-1 and 210 mmol/L hydrochloric acid, and 100 μL of calcium dye containing 78 μmol/L CPC, 3.36 mmol/L hydroxy-8-quinoline, and 25 mmol/L hydrochloric acid. The absorbance of the mixture was measured at 550 nm in a spectrophotometer (Synergy HTX Multi-Mode Microplate Reader, BioTek, Winooski, VT, USA). The absorbance values were expressed to calcium concentration by means of a calibration curve after subtraction of blank values of scaffolds in medium without cells. All samples were analyzed in quadruplicates of three independent experiments (n = 12).

#### 2.5.7. Collagen Production

Collagen type I is highly expressed by osteoblasts and involved in the formation of the ECM, where mineralization occurs. The collagen secretion and accumulation in the ECM of pre-osteoblastic cells cultured on different scaffolds were measured using a modified colorimetric assay [56] based on the Sirius Red staining method after 7, 14 and 21 days of culture. Briefly, at each experimental time point, 25 μL of culture medium were diluted in 75 μL of water and mixed with 1 mL 0.1% Direct Red 80 (Sigma-Aldrich, Burlington, MA, USA) dye in 0.5 M acetic acid and then incubated for 30 min at room temperature. After centrifugation of samples at 15,000× *g* for 20 min at 4 °C, the scaffolds were washed with 0.5 M acetic acid to remove the non-bound dye. The samples were centrifuged at 15,000× *g* for 20 min at 4 °C and dissolved in 1 mL 0.5 M NaOH. 200 μL of each solution were transferred to a 96-well plate and read at 530 nm. The absorbance values were translated to μg/mL of collagen by means of a calibration curve. All samples were analyzed in quadruplicates of three independent experiments (n = 12).

### 2.6. Statistical Analysis

Statistical analysis was performed for the assessment of the scaffolds’ compressive modulus, cell viability, ALP activity, calcium production, and collagen secretion using the one-way ANOVA Dunnett’s multi-comparison test in GraphPad Prism version 8 software (GraphPad Software, San Diego, CA, USA), comparing each composite with the pure polymer blend filament or scaffold, as well as comparing the two composites, at each experimental time period. *p*-values indicate statistically significant differences between the pure blend and the substituted composites (* *p* < 0.05, ** *p* < 0.01, *** *p* < 0.001, **** *p* < 0.0001, ***** *p* < 0.00001), as well as between the two composites (### *p* < 0.001).

## 3. Results

### 3.1. Characterization of Filaments

Polymer blend filaments incubated in PBS at 50 °C for eight weeks showed no significant mass loss (measured mass changes over the period within ±3% compared to mass at day 0), with no acidic degradation products detected in the weekly change of PBS (pH in the range 6.8 to 7.1 over the test period).

### 3.2. Characterization of Scaffolds

**Figure 2:**
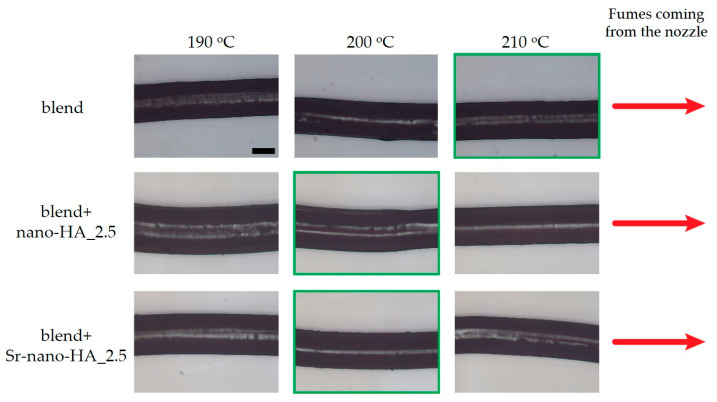
Optical microscopy images of the extruded strands during the preliminary extrusion experiments. In green, the final chosen temperature for each composition. Scale bars in the first top image represent 250 µm (for reference, same for all images).

Figure 2 highlights representative optical microscope images of the extruded filaments for each composition and temperature combinations.

**Figure 3:**
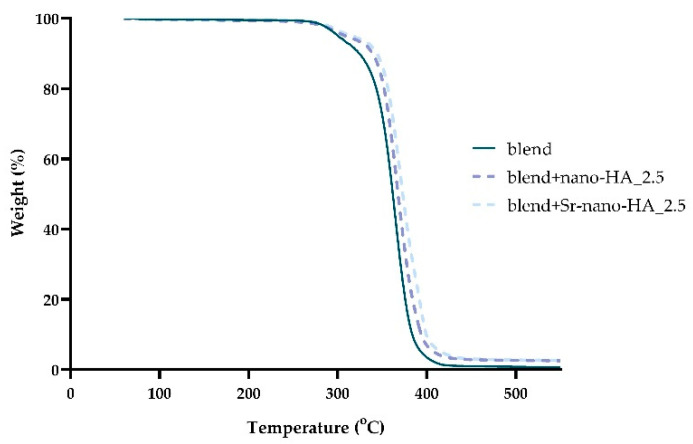
TGA curves of blend, blend+nano-HA_2.5 and blend+Sr-nano-HA_2.5 scaffolds.

The thermal properties of the scaffolds, the nano-HA, and Sr-nano-HA content were evaluated by TGA (Figure 3). The main weight loss of the blend was observed in the range of 280–400 °C due to the decomposition of the polymeric phases of PLLA, PCL and PHBV as previously described in PLLA:PCL [60] and PLLA:PHBV [24] scaffolds. Moreover, the thermal stability of the blend is slightly affected from the presence of PCL and PBHV, since pure PLLA is thermally stable until 390 °C [61]. It is known that the HA phase is thermally stable until 1300 °C [62]. Thus, the remaining mass of 2.5 and 2.6%wt above 390 °C corresponds to the inorganic phase nano-HA and Sr-nano-HA respectively, confirming their initial substitution degree.

**Figure 4:**
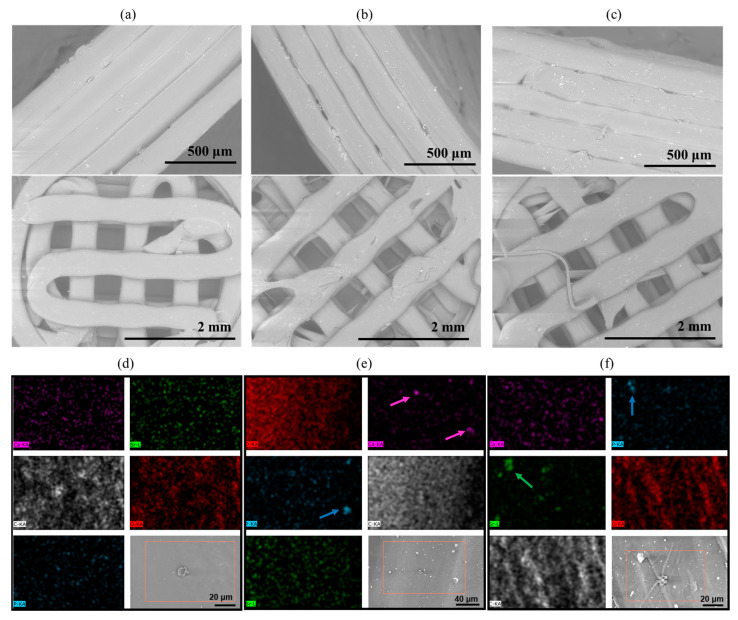
Representative SEM micrographs and EDS mapping of 3D scaffolds made of (**a**,**d**) blend, (**b**,**e**) blend+ nano-HA_2.5, and (**c**,**f**) blend+ Sr-nano-HA_2.5. Mapping colours indicating calcium (pink), phosphorus (blue), strontium (green), carbon (white), and oxygen (red). Arrows indicating nano-HA particles detected through a high number of counts of the respective element.

The morphology of the printed scaffolds was assessed by means of SEM (Figure 4a–c), which showed a smooth material surface and aligned layers, confirming the correctness of the printing parameters for the manufacturing of these materials. At higher magnification it is possible to observe the presence of the nanoparticles in the composite scaffolds, which are homogeneously distributed throughout the structure, without visible agglomerates.

The EDS analysis (Figure 4d–f) mainly detected carbon (C) and oxygen (O) for all samples, as expected, which are both associated with the polymer matrix. Only the composite scaffolds (Figure 4b,c) showed an evident presence of calcium (Ca) and phosphorus (P), evidenced by the colored arrows. As per the presence of Sr^2+^, this was only detected in the composite scaffold containing Sr-nano-HA (Figure 4c), as expected. The overall amount of each element in the investigated area of the scaffold is represented in Table 3 as atomic weight %. Detected values below 0.1 wt.% visible from the sample maps were considered background and are attributed to the semi-quantitative nature of this technique.

**Figure 5:**
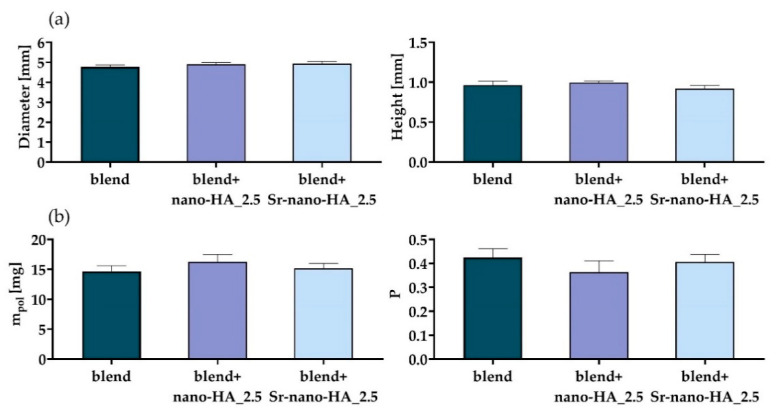
The measured scaffold diameter and height (**a**), and the measured mass (m_pol_) and estimated porosity (P) (**b**).

The results for the scaffold characterization procedure are reported in Figure 5, in terms of the measured diameter and height (Figure 5a), and of the measured mass and estimated porosity (Figure 5b).

**Figure 6:**
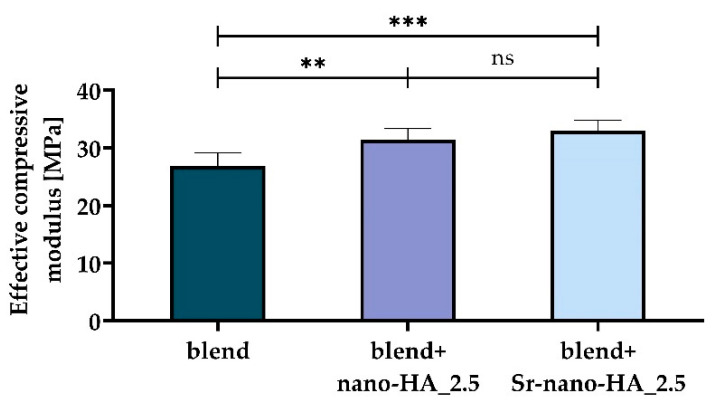
Comparison of the effective compressive modulus for the scaffold printed with the three tested polymer compositions. Statistically non-significant (ns, *p* ≥ 0.05) difference, ** *p* < 0.01, *** *p* < 0.001.

As can be seen from Figure 5, all measured dimensions are close to the designed ones and with a standard deviation which is 0.1 mm or lower for all groups. Regarding the scaffold internal porosity, the maximum standard deviation is around 5%. The main results from the scaffold mechanical characterization are shown in Figure 6. The mechanical behavior is linear in the tested range (data not shown). Furthermore, the effective compressive modulus (Figure 6) is significantly increased with the addition of both inorganic phases (*p*-value for the blend vs. blend+nano-HA_2.5 comparison lower than 0.01; *p*-value for the blend vs. blend+Sr-nano-HA_2.5 comparison lower than 0.001), while no difference has been found for the comparison blend+nano-HA_2.5 vs. blend+Sr-nano-HA_2.5 (*p*-value at 0.41). This is in accordance with previous literature reports [4].

### 3.3. Cell Viability and Proliferation in Direct Contact with the Extruded Filament Pieces

**Figure 7:**
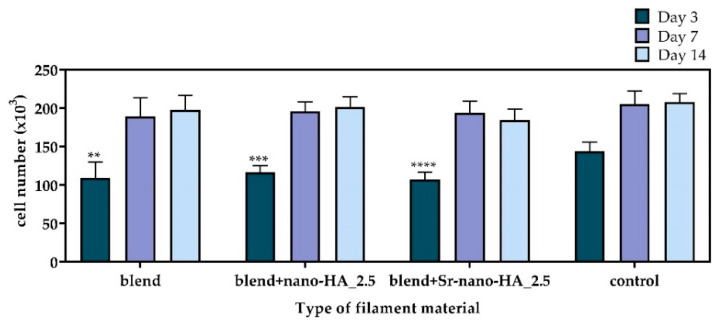
Viability and proliferation assessment showing the number of pre-osteoblastic cells cultured on the TCPS control and with polymeric blend and composite blend filaments after 3, 7, and 14 days. Each bar represents the mean ± SD of triplicates of three independent experiments (n = 12). Statistical analysis was performed for each material in comparison with the TCPS control (** *p* < 0.01, *** *p* < 0.001, **** *p* < 0.0001).

The cytotoxicity of the polymeric and composite materials was first assessed in the form of filaments by means of the PrestoBlue^TM^ reagent after 3, 7 and 14 days (Figure 7). The cell viability on the TCPS control surface on day 3 was significantly higher compared to the blend and the substituted blend materials. However, no differences were observed after 7 and 14 days, demonstrating the excellent cytocompatibility of all tested compositions. From day 3 to day 7 an increased cell proliferation is observed followed by a similar cell number on day 14, indicating a saturation in cell growth within the surface of the wells.

**Figure 8:**
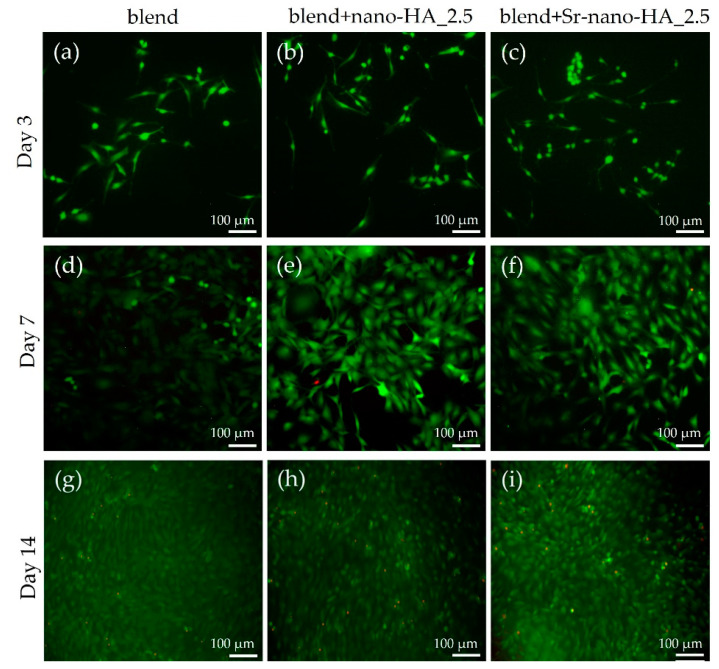
Live-dead assay images showing the viability of pre-osteoblastic cells cultured in the presence of extruded filaments comprising the pure polymeric blend and substituted blends with nano-HA and Sr-nano-HA after 3, 7 and 14 days. On day 3, only living cells are visible (in green) in the polymeric blend (**a**), blend+nano-HA_2.5 (**b**), and blend+Sr-nano-HA_2.5 (**c**). A higher number of living cells are observed on day 7 compared to day 3 in all three material compositions (**d**–**f**). After 14 days, a low number of dead cells (in red) are present compared to living cells for all three compositions (**g**–**i**). Scale bars represent 100 μm.

Moreover, the cell viability was assessed by means of live-dead staining and imaging. Cells cultured in direct contact with the filaments for 3, 7 and 14 days were stained with the live-dead assay and visualized under a fluorescence microscope (Figure 8). A significantly higher number of living cells depicted in green was observed in all filament compositions from day 3 to day 7 and up to day 14, while only a few dead cells were visualized in red. Specifically, on day 3, only living cells are visible in green in the pure polymeric blend (Figure 8a), blend+nano-HA_2.5 (Figure 8b) and blend+Sr-nano-HA_2.5 (Figure 8c). Similar results are observed on day 7 of culture in all three compositions as depicted in Figure 8d–f, indicating a proliferation increase compared to day 3. After 14 days, the majority of the cells are alive as per Figure 8g–i.

### 3.4. Evaluation of Cellular Responses on Scaffolds

#### 3.4.1. Cell Morphology

**Figure 9:**
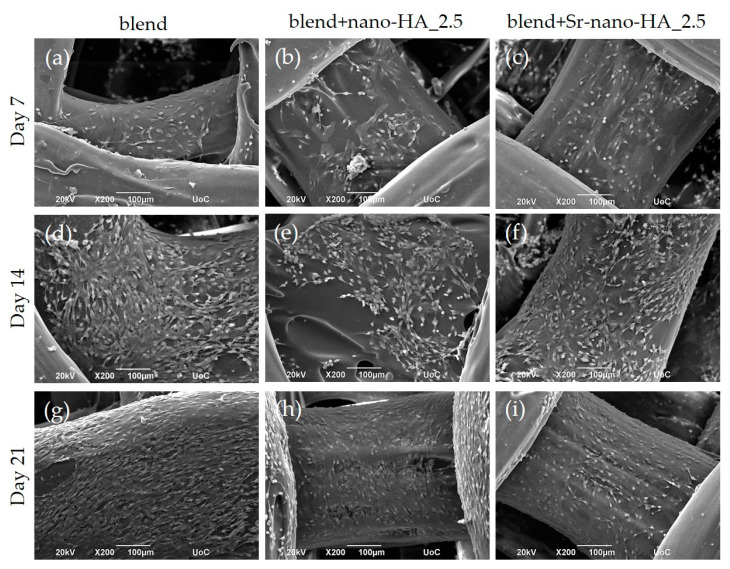
SEM images showing the morphology and adhesion of pre-osteoblastic cells cultured on polymeric blend and composite scaffolds for 7, 14, and 21 days. The images illustrate cell adhesion and growth on the polymeric blend and composite scaffolds after 7 days (**a**–**c**); after 14 days (**d**–**f**); and after 21 days (**g**–**i**). Original magnifications are ×200 and scale bars represent 100 μm.

The pre-osteoblastic cells were cultured on the scaffolds for 7, 14 and 21 days and observed by SEM. Representative SEM images (Figure 9) demonstrate an excellent cell attachment and growth within the scaffolds, with an increasing proliferation from day 7 to day 14 and up to day 21 in culture. The cells retain their characteristic elongated fibroblast-like morphology, forming intercellular connections on all three material compositions without visible differences. The surface of the composite scaffolds is visible on day 7 due to the initially low cell seeding density, which is gradually covered by a dense layer of cells after 14 and 21 days.

#### 3.4.2. Cell Viability and Proliferation

**Figure 10:**
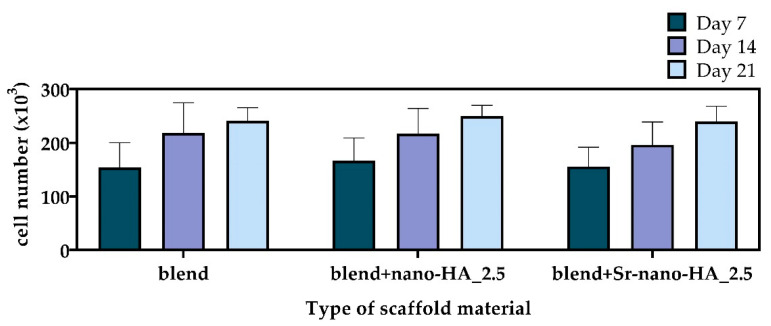
Viability and proliferation assessment showing the number of pre-osteoblastic cells on polymeric and composite scaffolds in osteogenic medium after 7, 14, and 21 days. Each bar represents the mean ± SD of triplicates of three independent experiments (n = 12). Statistical analysis was performed for each composite compared to the pure blend, as well as between the two composites, indicating non-significant differences among the scaffold compositions.

The cell viability of pre-osteoblastic cells on scaffolds was quantitatively assessed on days 7, 14 and 21 post seeding (Figure 10). At all experimental time points the number of viable cells on the polymeric blend and composites was similar, without significant differences among the different compositions. The number of living cells doubled on day 7 compared to the initially seeded cell number. In all three compositions the number of living cells increased from day 7 to day 14 and up to day 21, though not significantly, indicating that cells may have initiated differentiation and reduced proliferation. These results point out that all materials promote the viability and proliferation of the pre-osteoblastic cells, while the addition of the osteogenic medium promotes cell differentiation.

#### 3.4.3. Evaluation of the Osteogenic Potential of Scaffolds

##### ALP Activity

**Figure 11:**
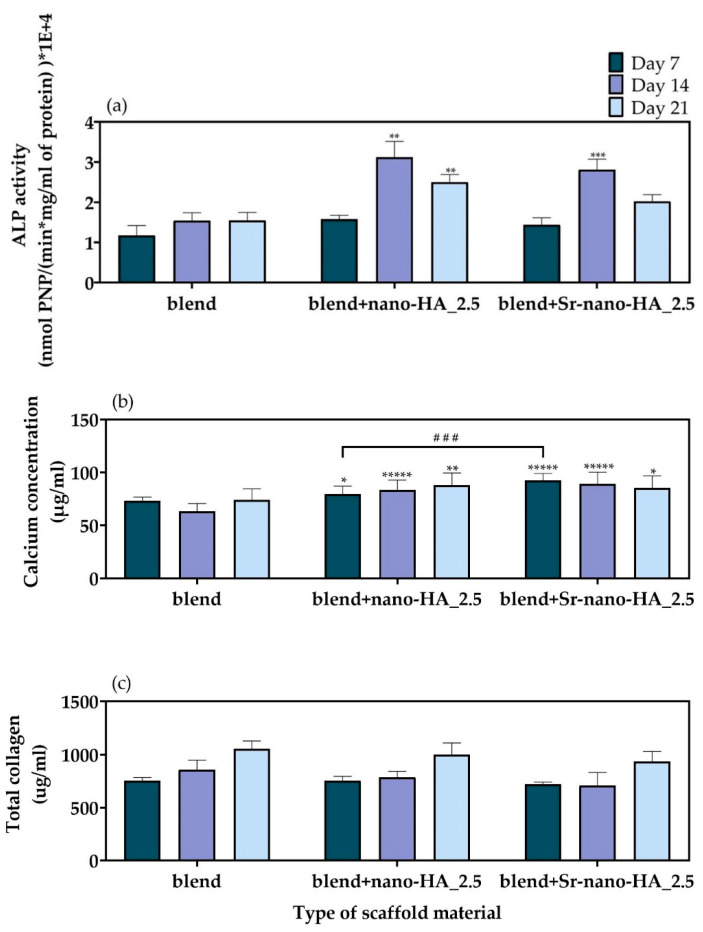
(**a**) Normalized ALP activity of the pre-osteoblastic cells cultured on polymeric blend, blend+nano-HA_2.5, and blend+Sr-nano-HA_2.5 scaffolds for 7, 14, and 21 days, (**b**) calcium concentration produced by the pre-osteoblastic cells cultured on polymeric and composite scaffolds after 7, 14, and 21 days, and (**c**) levels of collagen in the supernatants of pre-osteoblasts cultured on polymeric and composite scaffolds after 7, 14 and 21 days. Each bar represents the mean ± SD of triplicates of three independent experiments (n = 12) for each assay. Statistical analysis was performed for each composite compared to the pure blend scaffolds (* *p* < 0.05, ** *p* < 0.01, *** *p* < 0.001, ***** *p* < 0.00001), as well as between the two composites (### *p* < 0.001). For the total collagen quantification (**c**), no statistically significant differences among the scaffold compositions have been observed.

The results of the ALP activity of the pre-osteoblastic cells cultured for 7, 14, and 21 days on polymeric blend and composite scaffolds are shown in Figure 11a. The cells seeded onto the polymer blend scaffold produced the same amount of ALP throughout the study, whilst for composite samples the ALP concentrations increased between day 7 and day 14. This suggests the onset of the cell differentiation process which continued up to day 21 for both composites. Moreover, both composites reported a slight decrease in ALP production between day 14 and day 21 inferring that the cell differentiation in these samples is slowing down, often associated with the start of the mineralization process [44].

##### Calcium Production

Calcium produced by the pre-osteoblastic cells cultured on the blend, blend+nano-HA_2.5, and blend+Sr-nano-HA_2.5 scaffolds was assessed after 7, 14 and 21 days. The results shown in Figure 11b show that the amount of calcium produced by cells on the polymer blend scaffold did not change significantly with time. Instead, a significantly higher calcium concentration was found for the composites compared to the pure blend scaffolds at all experimental time points. The calcium concentration shows a slight increase on blend+nano-HA_2.5 scaffolds and a slight decrease on blend+Sr-nano-HA_2.5 as the culture period proceeds. Moreover, the Sr-nano-HA substituted blend scaffolds show significantly higher calcium production compared to the nano-HA composite scaffolds on day 7 of culture.

##### Collagen Secretion

The secreted total collagen concentration in supernatants was measured after 7, 14 and 21 days in culture (Figure 11c). The produced collagen was found to be higher on both composite scaffolds compared to the polymeric blend scaffolds, particularly on day 21. However, this was not to a significant level. The levels of extracellular collagen are high from the first week in culture, indicating a healthy ECM formation, which is necessary for the bone remodeling process.

## 4. Discussion

Biomaterials in BTE act as matrices to support cell viability, proliferation, osteogenic differentiation and ECM formation, an orchestrated process resulting in bone regeneration. In this study, PLLA, PCL and PHBV were blended in an optimal ratio of 90/5/5 wt% to create filaments by extrusion. In addition, the blends PLLA/PCL/PHBV were combined with nano-HA or Sr-nano-HA due to their osteoconductive properties, to create composite filaments.

Relevant FDM printing parameters (i.e., extrusion and bed temperature, printing speed) were optimized for each filament composition. The results from this characterization highlighted that all materials have similar printing parameters except for the bed temperature, which was chosen much higher for the blend filament to increase adhesion, easily seen from the SEM analysis of the produced scaffolds. This is probably related to the fact that the incorporation of inorganic phases may increase the time for the temperature to set, avoiding warping due to rapid cooling. The optimized set of printing parameters was used to produce 3D porous scaffolds with high shape fidelity. Porosity is particularly important for a BTE scaffold, since open and interconnected pores of the proper size are essential to provide cells with proper nutrient diffusion, waste removal, and housing. Here, we obtained a high porosity close to the desired one (with designed pores of 800 µm), which has been shown to promote bone formation while preventing the development of fibrous tissue [4]. The compressive modulus values of 27 MPa for the polymer blend scaffolds and 32 MPa for the substituted with nano-HA and Sr-nano-HA scaffolds present mechanically suitable structures for the regeneration of cancellous bone which has a compressive strength of 4–12 MPa [63]. Similar results have been reported in the literature indicating that the combination of nano-HA with polymeric materials enhances the mechanical properties of the material and retaining their bioactivity [63]. Our results on the polymer blend filaments degradation showed a non-significant mass loss within 3% over the time period investigated in this study, indicating that their degradation will take a longer time. PLA degradation is well described [50], but PLLA/PCL/PHBV is a new polymer blend. The longer-term degradation of the blend scaffolds will be studied in future work to show how the concentrations of PCL and PHBV would affect their degradation rate. Scaffolds that degrade at the same rate or slower than it takes for the new bone tissue to form are beneficial. In the literature, degradation of PLLA/HA and PLLA/Sr-HA scaffolds with a mass loss of 4% after four weeks have been reported to have considerable potential for bone repair [49]. Moreover, PLA/HA scaffolds with a mass loss lower than 1% after four weeks have been described to promote the osteogenic differentiation of human mesenchymal stem cells in the absence of osteogenic stimuli [64]. In the human body, PLLA-based scaffolds degrade through hydrolysis and enzymatic degradation. The immune cells secrete enzymes such as acid phosphatase and lactate dehydrogenase, which may enhance the PLLA degradation [65].

The FDM printed scaffolds were evaluated for their osteogenic potential in vitro. The aim was to combine the toughness of PLLA/PCL/PHBV blend with the inorganic phase of nano-HA and Sr-nano-HA to improve the mechanical properties [66] and the degradation profiles, and to introduce osteoconductive properties. The addition of the appropriate amount of nano-HA induces the osteogenic potential of the polymeric materials [66] while a 3D porous microenvironment is a crucial component for bone regeneration. The degradation of PLLA leads to the release of acidic by-products that can affect cellular responses [30]. Nano-HA buffers these products and may block unfavorable cell damage. In addition to nano-HA, Sr-nano-HA has also been investigated. Sr^+2^ is a trace element in the human body, which has been extensively studied for the treatment of osteoporosis [67]. Recent studies have shown that the addition of Sr^+2^ enhances bone formation while in parallel suppressing bone resorption [68].

For the in vitro investigation of the osteogenic potential of the different scaffold compositions, pre-osteoblastic cells were cultured within the scaffolds for three weeks and the osteogenic markers, including the ALP activity and the calcium and collagen production, were monitored weekly. The cell viability assessment displays that all three material compositions have excellent biocompatibility allowing cells to proliferate. This finding has been confirmed by the SEM images, revealing that the cells are well-spread and depict a physiological morphology from the first time of observation for all three compositions with increasing cell proliferation. Previous studies on PHBV/PLLA scaffolds substituted with nano-HA [24], on pure PCL/PLLA electrospun matrices [23], and PCL with Sr-nano-HA scaffolds [26] showed comparable results and materials with increased biocompatibility.

Nano-hydroxyapatite is essential for bone regeneration, as its incorporation in polymeric materials increases their mechanical strength and osteoconductivity [69]. It is known that calcium contained in the nano-HA crystals and strontium are physiologically similar, and that the latter is incorporated into bones by ionic exchange with calcium [70]. The role of strontium in bone metabolism is well described. In postmenopausal women, strontium is administered as strontium ranelate to decrease the risk of bone fracture since it induces the action of osteoblasts while in parallel inhibiting the action of osteoclasts [71]. The presence of Sr^2+^ in the composite scaffolds containing Sr-nano-HA was confirmed by EDS, demonstrating for both composites well distributed nanoparticles within the polymer matrix, being accessible to the cells by direct contact. Additionally, the percent substitution of the inorganic phase into the polymeric matrix was confirmed by the TGA analysis. To assess the scaffolds capacity on pre-osteoblastic cell differentiation, the osteogenic potential of pure blend and substituted 3D printed scaffolds was evaluated by quantification of the ALP activity, calcium production and secreted collagen. The ALP activity is one of the most well-studied early markers and calcium production is one of the late markers of osteogenesis. The results indicated that the materials are cytocompatible. The fact that the cell number is similar in all three experimental time points indicates that cells may prefer to differentiate instead of proliferating as they are cultured in the presence of osteogenic medium. After 14 days in culture, the ALP activity is almost two-fold higher compared to day 7 on the blend+nano-HA_2.5 and blend+Sr-nano-HA_2.5, followed by a decrease. The ALP activity in the blend scaffolds shows similar levels at all time points, demonstrating that the addition of nano-HA and Sr-nano-HA promotes the osteogenic differentiation on day 14. Previous studies revealed similar responses in PLLA, PCL and PHBV materials, pure or in combination with nano-HA or Sr-nano-HA [23,24,26,27]. The calcium concentration results are in accordance with the ALP activity data. Both composites have significantly higher calcium concentration compared to the pure blend at all time points, and this is in line with relevant reports [22,23,24,27]. On day 7, the Sr-nano-HA substituted blend scaffolds show significantly higher calcium production compared to the nano-HA composite scaffolds. High concentrations of collagen from the first week in culture on all three scaffold compositions are important towards ECM formation, which is essential for bone remodeling. In conclusion, our results indicate the initiation of osteoblast formation within the scaffolds as evidenced from the ALP activity, the calcium production data on day 14, and the high values of produced collagen on day 21. These results are in agreement with previous literature findings [72] suggesting that the incorporation of the inorganic components of nano-HA and Sr-nano-HA enhances the osteogenic potential of the polymeric materials. The incorporation of Sr-nanoHA into a polymeric matrix affects the release rate and the availability of the inorganic components, and thus the effectiveness of the cellular responses, as previously reported [36,44]. Recently, a sustained release of strontium ions of 0.5 μg/mL after 7 days from composite PLLA-Sr-nanoHA filaments containing 50% of Sr^2+^ substitution in Sr-nanoHA has been presented [36]. In another study on PLA scaffolds substituted with 20 and 25% of nano-HA, the authors showed 1% of the calcium ions and less than 2% of the phosphate ions being released after 4 weeks in culture [64]. These levels of ion release from the PLA-HA scaffolds, and a degradation rate of 1.5% mass loss after ten weeks, have been reported as adequate to induce the osteogenic differentiation in vitro. Another work on PLLA/Sr-HA scaffolds fabricated by selective laser sintering indicated a sustained release of strontium ions with a cumulative concentration of 5.6 μg/mL over four weeks [49]. Moreover, this report shows similar levels of the cumulative release of calcium ions from both PLLA/HA and PLLA/Sr-HA composite scaffolds, without any significant differences. This observation may explain why we did not find significant differences in the biological experiments between the two investigated composite scaffolds in this study. Our findings demonstrate that both composite scaffolds promote the osteogenesis in vitro. Their implantation into cranial defects in ovariectomized mice to show in vivo new bone formation in an osteoporotic model is subject of a future work. Reports on the implantation of 3D printed PLA-HA composite scaffolds in rabbits indicated their effectiveness on repairing bone defects [73,74]. Moreover, the implantation of composite scaffolds comprising PLLA with mineralized strontium-doped hydroxyapatite in rabbits has been reported to promote bone regeneration [51].

## 5. Conclusions

In this study, novel three-component polymer blends comprising PLLA/PCL/PHBV were enriched with nano-HA and Sr-nano-HA to produce composite filaments. The ratio of the polymers has been previously optimized, with the 90/5/5 wt% PLLA/PCL/PHBV composition indicating the best thermal and mechanical properties, thus selected for biological evaluation in BTE applications. The blend with the incorporation of the inorganic phase nano-HA and Sr-nano-HA was evaluated in the form of extruded filaments in a pre-osteoblastic cell culture. The results show absence of any cytotoxic effects for 14 days. Three-dimensional scaffolds have been printed by FDM from PLLA/PCL/PHBV blend and composite filaments and evaluated mechanically and biologically for their capacity to support bone formation in vitro. The scaffolds indicated a mean porosity of 40%, mean pores of 800 µm, with an effective compressive modulus of 27 MPa for the blend scaffolds and 32 MPa for the composite scaffolds. Moreover, the 3D printed scaffolds have been evaluated for their biocompatibility and osteogenic potential in a pre-osteoblastic cell culture. The results confirm that the blend scaffolds and their composite counterparts are biocompatible with high proliferation capacity. Interestingly, the incorporation of the osteoinductive materials nano-HA and Sr-nano-HA into the blend scaffolds showed significantly higher levels of ALP activity and calcium production compared to the blend scaffolds. These results demonstrate that both composites have excellent mechanical properties and high osteogenic capacity, and can be applied in BTE applications.

## Figures and Tables

**Table 1:** Measured mean diameter and standard deviation for the filaments of the three material compositions, polymer blend (blend), and blend substituted with 2.5% nano-HA or Sr-nano-HA.

Filament Composition	Mean Diameter [55]	Standard Deviation [55]	Number of Measurements
blend	1.87	0.08	12
blend+nano-HA_2.5	1.72	0.08	12
blend+Sr-nano-HA_2.5	1.62	0.1	12

**Table 2:** Final set of printing parameters chosen from the preliminary printing tests and used to produce all scaffolds.

Filament Composition	Printing Speed [mm/s]	Extrusion Temperature [°C]	Bed Temperature [°C]	Layer Height [55]
blend	10	210	80	0.2
blend+nano-HA_2.5	10	200	45	0.2
blend+Sr-nano-HA_2.5	10	200	45	0.2

**Table 3:** Distribution of elements in the printed samples (atomic weight %), detected with EDS. The presented values do not amount to 100% due to the SD error associated with the measurements.

Element	Blend (at wt. %) Mean ± SD	blend+nano-HA_2.5 (at wt. %) Mean ± SD	blend+Sr-nano-HA_2.5 (at wt. %) Mean ± SD
Carbon	57.19 ± 0.64	54.44 ± 1.36	58.24 ± 0.68
Oxygen	42.73 ± 0.66	44.87 ± 1.61	40.05 ± 1.14
Strontium	0.05 ± 0.03	0.06 ± 0.07	0.92 ± 0.60
Phosphorus	0.02 ± 0.02	0.17 ± 0.06	0.23 ± 0.14
Calcium	0.01 ± 0.01	0.46 ± 0.16	0.56 ± 0.48

## Data Availability

The data presented in this study are openly available in ZENODO: https://doi.org/10.5281/zenodo.7299841 (accessed on 7 November 2022).
